# The value of nanopore sequencing as a diagnostic tool in tuberculous meningitis: A protocol of systematic review and meta-analysis

**DOI:** 10.1371/journal.pone.0307389

**Published:** 2024-07-18

**Authors:** Yanqin Shen, Yuyang Ling, Guocan Yu, Xiaoxia Zhang

**Affiliations:** 1 Department of Nursing, Hangzhou Red Cross Hospital, Hangzhou, Zhejiang, China; 2 Zhejiang Chinese Medical University, Hangzhou, Zhejiang, China; 3 Zhejiang Tuberculosis Diagnosis and Treatment Center, Hangzhou Red Cross Hospital, Hangzhou, Zhejiang, China; The University of Georgia, UNITED STATES OF AMERICA

## Abstract

**Background:**

Rapid diagnosis of tuberculous meningitis (TBM) remains very difficult. Nanopore sequencing is gaining ground in the field of rapid tuberculosis (TB) diagnostics. The purpose of this study was to complete a protocol to guide the conduct of a systematic review and meta-analysis evaluating the accuracy of nanopore sequencing for the rapid diagnosis of TBM.

**Methods:**

In accordance with the Preferred reporting items for systematic review and meta-analysis protocols (PRISMA-P) guidelines, we completed this protocol, which was also registered on the PROSPERO platform. We will search the EMBASE, PubMed, the Cochrane Library, Wanfang database, and China National Knowledge Infrastructure databases for literature that evaluated the accuracy of nanopore sequencing for rapid diagnosis of TBM and screen them according to the inclusion and exclusion criteria, and qualified literature will be extracted with relevant data for further analysis. Quality Assessment of Diagnostic Accuracy Studies (QUADAS-2) will be used for evaluating the methodological quality of included studies. Stata (V 15.0; Stata Corp., College Station, TX, the USA) with *midas* module will be used to perform relevant meta-analysis. Heterogeneity between studies will be assessed by *I*^2^ statistics. When significant heterogeneity exists between studies, we will conduct meta-regression analyses, subgroup analyses and sensitivity analyses to further explore the sources of heterogeneity.

**Conclusion:**

We completed this study protocol, and this systematic review and meta-analysis will be the first systematic evaluation of the role of nanopore sequencing in the rapid diagnosis of TBM, which will allow clinicians to have a better understanding of the test.

**Trial registration:**

Systematic review registration PROSPERO Registration number: CRD42024549837.

## Introduction

Tuberculosis (TB), an infectious disease caused by human infection with *Mycobacterium tuberculosis* (MTB), is an ancient disease that threatens human health [1]. TB is one of the major sources of death from a single infectious disease [2]. The most common location of MTB infection is the lungs, known as pulmonary TB (PTB), while extrapulmonary TB (EPTB) occurs when the pathogen infects organs other than the lungs [3]. Tuberculous meningitis (TBM) is a critical type of EPTB, accounting for only 1–5% of all new cases, but the consequences are devastating, leading to severe disability or death in about half of patients [4]. The main reason for this outcome is that early diagnosis of TBM is very challenging, and delays in treatment for lack of diagnosis ultimately lead to disease progression and poor prognosis [5]. Improving the ability to rapidly diagnose TBM at an early stage is essential for this disease.

Classical MTB screening techniques are hardly up to the task of rapid diagnosis of TBM [6]. The acid-fast bacilli (AFB) smear is the most widely used MTB screening method, with a low entry barrier to conduct and inexpensive, but its detection ability in cerebrospinal fluid (CSF) is very low and most TBM will be missed [7]. MTB culture is the gold standard for TB diagnosis, but its sensitivity in TBM is unsatisfactory, while it takes weeks to obtain results and does not meet the requirements for rapid diagnosis [8]. The rapid development of molecular biology techniques has made possible the rapid diagnosis of infectious diseases [9]. Nucleic acid amplification tests (NAATs) represented by Xpert MTB/RIF have shown strong advantages in the field of rapid diagnosis of TB, and the rapid diagnosis of TB is becoming more and more efficient [10,11]. However, the application of these NAATs in TBM is not as effective as in other types of TB such as PTB and lymph node TB [12].

The widespread use of genome sequencing is an important event in the field of rapid diagnosis of TB, which has led to new competent tools for the diagnosis of TB [13,14]. The diagnostic sensitivity of next-generation sequencing (NGS) in TBM still has room to rise [15,16]. Nanopore sequencing is a new generation sequencing method, which has the advantages of long reads and real-time detection compared with NGS [17]. Nanopore sequencing is becoming more and more widely used in the field of TB [18,19], gaining recognition from clinicians, and the method may be a new star in the rapid diagnosis of TBM. There is a lack of evidence-based medical evidence for the rapid diagnosis of TBM by nanopore sequencing; therefore, the purpose of this study was to complete a protocol to guide the conduct of a systematic review and meta-analysis evaluating the accuracy of nanopore sequencing for the rapid diagnosis of TBM.

## Methods

### Design and registration

Prior to reporting the final results of the systematic review and meta-analysis, we designed and completed the protocol for this meta-analysis in accordance with the Preferred reporting items for systematic review and meta-analysis protocols (PRISMA-P) statement [20] and registered it on the PROSPERO platform under the registration number CRD42024549837. PRISMA-P checklist was shown in S1 File. We will rely on the Preferred Reporting Items for a Systematic Review and Meta-analysis of Diagnostic Test Accuracy Studies: The PRISMA-DTA Statement for final results reporting when the study is completed [21]. This study will be conducted based on public databases and does not require ethical approval.

### Information sources

Embase, Cochrane Library, PubMed, Wanfang Database, and China National Knowledge Infrastructure will be used to search for potentially eligible studies in June 2025. References cited in meta-analysis or reviews on related topics will also be assessed for inclusion in this study.

### Search strategy

Yanqin Shen and Yuyang Ling collaborated to design search strategies for relevant databases. We will not impose language restrictions when performing database searches.

The search strategy used in PubMed is as follows:

#1 "Tuberculosis, Meningeal"[Mesh] OR “Meningeal Tuberculoses” OR “Meningeal Tuberculosis” OR “TB Meningiti*” OR “Tubercular Meningiti*” OR “Meningiti*, Tubercular” OR “Meningiti*, Tuberculous” OR “Tuberculous Meningiti*” OR “Meningiti*, Tuberculosis” OR “Tuberculosis Meningiti*”

#2 “Extrapulmonary tuberculosis” OR “Extra pulmonary tuberculosis” OR EPTB

#3 #1 OR #2

#4 "Nanopore Sequencing"[Mesh] OR “Nanopore Sequencings” OR “Sequencing, Nanopore” OR “third generation sequencing” OR “Oxford Nanopore Technolog*” OR ONT

#5 #3 AND #4

Search strategies for other Chinese and English databases were shown in S2 File.

### Eligibility criteria

#### Type of study

Any study that evaluated the accuracy of nanopore sequencing for the diagnosis of TBM will be included, regardless of the type of study.

#### Patients

The patients enrolled will be those with suspected TBM, regardless of gender, race, or age.

#### Index tests

Nanopore sequencing will be determined to be index test.

#### Comparative test

Comparative tests will be not required. This study will not compare nanopore sequencing to other diagnostic tests.

#### Outcomes

Diagnostic accuracy indicators such as sensitivity, specificity, positive predictive value (PPV), negative predictive value (NPV), area under the curve (AUC) will be treated as outcomes.

#### Reference standards

MTB culture and clinical diagnosis will be used as reference standards. Clinical diagnosis was determined after a comprehensive evaluation of the patient’s clinical symptoms, imaging findings, TB immunoassay results, CSF biochemistry results, AFB smear, culture, results of other NAATs tests, and the efficacy of antituberculosis treatment.

### Target conditions

Original studies that used well-defined reference standards and reported values for true positive (TP), false positive (FP), false negative (FN), and true negative (TN), or that four values could be calculated from available information will be included. When information provided in an article is incomplete, the corresponding author will be contacted for additional information. Exclusion criteria will be TP, FP, FN.

TN values are unavailable, case studies, sample size less than 10, reviews, meta-analysis, abstract only without full text, studies not published in Chinese or English.

### Literature screening and selection

We will use Endnote X9.2 for literature management, and literature obtained from individual database searches will be imported into Endnote first. Two independent researchers (Yanqin Shen and Yuyang Ling) will review the title, abstract and full text of the imported literature individually and retain or exclude relevant literature based on the inclusion and exclusion criteria with justification. When the two disagree, a third independent researcher (Xiaoxia Zhang) will join the discussion.

### Data extraction

After determining the final inclusion of the study, two independent researchers, the same as in the literature screening phase, will separately extract the relevant data from the study. The extracted data mainly included the name of the first author, year of publication, country, type of study, method of patient selection (consecutive or not), number of patients, type of patients (children or adults), HIV infection status, reference standard, type of specimen (CSF or other), method of specimen pretreatment, status of the specimen (fresh or frozen), type of nanopore sequencing (targeted amplification or not), and values of TP, FP, FN, TN. Obtained data will be cross-checked and inconsistencies will be double-checked and confirmed by a third researcher. If two different reference standards are included in a study, we will treat them as two separate studies based on the reference standards.

### Quality evaluation

We will use the Quality Assessment of Diagnostic Accuracy Studies (QUADAS-2) tool for quality evaluation of the included studies [22]. The quality of the studies will be evaluated and justified on a case-by-case basis according to the entries in the tool, and disputed cases will be resolved through a third party.

### Publication bias

According to the PRISIMA-DTA guidelines, DTA meta-analyses can be performed without the assessment of publication bias because the current methods for assessing publication bias are flawed [21].

### Evidence evaluation

We will use the Grading of Recommendations Assessment, Development, and Evaluation (GRADE) for the level of evidence evaluation. The level of evidence for each study will be categorized into high, moderate, low, and very low according to the guideline [23].

### Data synthesis and statistical analysis

We will first calculate the pooled sensitivity, specificity, PPV, NPV, AUC and their 95% confidence intervals using the four values obtained for TP, FP, FN, and TN, then plot the forest plots and the summary receiver operating characteristic curve. Different reference standards will be treated separately. Heterogeneity between studies will be assessed by *I*^2^ statistics, *I*^2^ = 0, suggesting no significant heterogeneity between studies and *I*^2^ > 50%, suggesting significant heterogeneity between studies. When significant heterogeneity was present, we will perform meta-regression analysis, subgroup analysis and sensitivity analysis to explore possible sources of heterogeneity. We will perform meta-regression analysis and subgroup analysis based on these parameters: type of study, method of patient selection, type of patients, HIV infection status, type of specimen, method of specimen pretreatment, status of the specimen, type of nanopore sequencing. Sensitivity analysis assesses whether a study is a high-risk study by removing the study and providing the results of the analysis with and without this study. Sensitivity analysis will be performed to assess the reliability and robustness of the aggregation results. Stata (V 15.0; Stata Corp., College Station, TX, the USA) with *midas* module will be used to perform relevant meta-analysis and plotting of summary receiver operating characteristic curves and forest plots, and it requires a minimum of four studies under each predefined variable to complete the statistical analysis [24].

## Discussion

Early and precise diagnosis and treatment of TBM can enable patients to receive accurate treatment and reduce the incidence of serious complications. However, it is extremely difficult to obtain early bacteriologic evidence of TBM [25]. Nanopore sequencing may be able to provide a high-value role in the early precision diagnosis of TBM. In order to fully evaluate the role of nanopore sequencing in the early diagnosis of TBM, we will conduct a systematic review and meta-analysis, which to the best of our knowledge should be the first one, before that, we have completed the protocol of this systematic review and meta-analysis and seek to publish it in a peer-reviewed journal, and through the peer-review, we will improve the protocol of this study to make the subsequent meta-analysis more standardized and effective.

## Supporting information

S1 File Preferred Reporting Items for Systematic review and Meta-Analysis Protocols (PRISMA-P) checklist. (DOC)

S2 File Search strategies for Chinese and English databases. (DOCX)
