# Supplementary material for: The value of nanopore sequencing as a diagnostic tool in tuberculous meningitis: A protocol of systematic review and meta-analysis
Source: PLoS One. 2024 Jul 18;19(7):e0307389. doi: 10.1371/journal.pone.0307389 (PMC11257307; doi:10.1371/journal.pone.0307389)
Supplement: S2 File — (DOCX) [file pone.0307389.s002.docx]

Pubmed and Cochrane

#1 "Tuberculosis, Meningeal"[Mesh] OR “Meningeal Tuberculoses” OR “Meningeal Tuberculosis” OR “TB Meningiti*” OR “Tubercular Meningiti*” OR “Meningiti*, Tubercular” OR “Meningiti*, Tuberculous” OR “Tuberculous Meningiti*” OR “Meningiti*, Tuberculosis” OR “Tuberculosis Meningiti*”

#2 “Extrapulmonary tuberculosis” OR “Extra pulmonary tuberculosis” OR EPTB

#3 #1 OR #2

#4 "Nanopore Sequencing"[Mesh] OR “Nanopore Sequencings” OR “Sequencing, Nanopore” OR “third generation sequencing” OR “Oxford Nanopore Technolog*” OR ONT

#5 #3 AND #4

EMBASE

#1 "Tuberculosis, Meningeal"[Mesh] OR “Meningeal Tuberculoses” OR “Meningeal Tuberculosis” OR “TB Meningiti*” OR “Tubercular Meningiti*” OR “Meningiti*, Tubercular” OR “Meningiti*, Tuberculous” OR “Tuberculous Meningiti*” OR “Meningiti*, Tuberculosis” OR “Tuberculosis Meningiti*”

#2 “Extrapulmonary tuberculosis” OR “Extra pulmonary tuberculosis” OR EPTB

#3 #1 OR #2

#4 "Nanopore Sequencing"[Mesh] OR “Nanopore Sequencings” OR “Sequencing, Nanopore” OR “third generation sequencing” OR “Oxford Nanopore Technolog*” OR ONT

#5 #3 AND #4

#1 'tuberculous meningitis'/exp OR 'Tuberculosis, Meningeal' OR 'Meningeal Tuberculoses' OR 'Meningeal Tuberculosis' OR 'TB Meningiti*' OR 'Tubercular Meningiti*' OR 'Meningiti*, Tubercular' OR 'Meningiti*, Tuberculous' OR 'Tuberculous Meningiti*' OR 'Meningiti*, Tuberculosis' OR 'Tuberculosis Meningiti*'

#2 ‘Extrapulmonary tuberculosis’ OR ‘Extra pulmonary tuberculosis’ OR EPTB

#3 #1 OR #2

#4 'Nanopore Sequencing' OR 'Nanopore Sequencings' OR 'Sequencing, Nanopore' OR 'third generation sequencing' OR 'Oxford Nanopore Technolog*' OR ONT

#5 #3 AND #4

CNKI AND Wanfang

#1 结核性脑膜炎 OR 脑脊液 OR 脑结核 OR脑膜结核 OR 肺外结核

#2 纳米孔测序 OR 三代测序 OR 牛津纳米孔

#3 #1 AND #2
